# Does resistance really carry a fitness cost?

**DOI:** 10.1016/j.cois.2017.04.011

**Published:** 2017-06

**Authors:** Richard H ffrench-Constant, Chris Bass

**Affiliations:** Centre for Ecology and Conservation, University of Exeter, Penryn Campus, Penryn, Cornwall TR10 9FE, UK

## Abstract

Insecticide resistance mutations are widely assumed to carry fitness costs. However studies to measure such costs are rarely performed on genetically related strains and are often only done in the laboratory. Theory also suggests that once evolved the cost of resistance can be offset by the evolution of fitness modifiers. But for insecticide resistance only one such example is well documented. Here we critically examine the literature on fitness costs in the absence of pesticide and ask if our knowledge of molecular biology has helped us predict the costs associated with different resistance mechanisms. We find that resistance alleles can arise from pre-existing polymorphisms and resistance associated variation can also be maintained by sexual antagonism. We describe novel mechanisms whereby both resistant and susceptible alleles can be maintained in permanent heterozygosis and discuss the likely consequences for fitness both in the presence and absence of pesticide. Taken together these findings suggest that we cannot assume that resistance always appears *de novo* and that our assumptions about the associated fitness costs need to be informed by a deeper understanding of the underlying molecular biology.

## Highlights

• New resistance mutations are predicted to be costly but this is rarely shown in the field.
• Fitness costs are predicted to be offset by ‘modifier’ loci but specific examples are rare.
• Resistance mutations can be pre-existing polymorphisms or maintained by sexual antagonism.
• Duplication of resistance loci can maintain a susceptible copy in permanent heterozygosis.
• CRISPR-CAS can be used to make mutations in a defined genetic background for future fitness studies.

**Current Opinion in Insect Science** 2017, **21**:39–46

This review comes from a themed issue on **Pests and resistance**

Edited by **Thomas W Sappington** and **Nicholas John Miller**

For a complete overview see the Issue and the Editorial

Available online 22nd May 2017

**http://dx.doi.org/10.1016/j.cois.2017.04.011**

2214-5745/Crown Copyright © 2017 Published by Elsevier Inc. This is an open access article under the CC BY license (http://creativecommons.org/licenses/by/4.0/).

Our ability to manage xenobiotic resistance (both to drugs and pesticides), relies on the ‘alternation’ (or ‘mixture’) of classes of compound with differing modes of action. Management strategies using such alternation of differing chemical classes assume that resistance to compound A will decline during the subsequent use of compound B. This assumption is based on the prediction that *de novo* resistance to compound A will carry a fitness cost and that the frequency of resistance to A will therefore decline while compound B (or no compound) is used instead. This assumption, that resistance carries a cost in the absence of the xenobiotic, is therefore central to current resistant management strategies in both agriculture (pesticide resistance) and medicine (antibiotic resistance and cancer tumour drug resistance). Despite the widespread reliance on such predicted fitness costs to decrease the frequency of xenobiotic resistance, and an ample literature on the subject, the documentation of such costs is in fact fraught with technical difficulty. Here we will focus our discussion on fitness costs associated with insecticide resistance but it is important to remember that such principles also apply to the management of resistance to all pesticides and drugs.

In the year 2000, Coustau *et al.* suggested that ‘fitness costs can only be fully interpreted in the light of the molecular mutations that might underlie them’ [1]. Here, some 17 years later, and following an explosion in the molecular analysis of insecticide resistance, we therefore now examine the extent to which this is true. Classical theory predicts that *de novo* mutations that confer resistance to pesticides should carry a fitness cost in the absence of pesticide. This theory is based on a model developed by Fisher [2] which suggests that independent selection pressures shape the present (almost) optimal phenotypes via complex gene coevolution. In view of this gene interdependence any new resistance associated mutation of major effect would therefore be predicted to be highly deleterious. Similarly, theory also suggests that once a new mutation has arisen then other loci within the genome can act as ‘modifiers’ to ameliorate the negative fitness costs associated with resistance in the absence of pesticide. However, as discussed below, well documented examples of such fitness modifiers are in fact very rare [3, 4]. Here we will therefore critically examine if the current body of literature supports the assumption that resistance always carries a cost. We will do this by addressing several fundamental questions. First, under what conditions can we realistically measure any potential fitness costs for different resistant strains? Second, what evidence is there that fitness costs are offset by the evolution of modifiers or are many resistance mutations in fact pre-existing polymorphisms with pleiotropic effects? Third, has the explosion of resistance associated molecular biology really helped us to understand when and where resistance might carry a cost?

## Counting the cost

Numerous case studies of fitness costs attributed to insecticide resistance have been recently and comprehensively reviewed elsewhere [5]. A review of this review suggests to us several basic rules for experiments designed to study the fitness costs of resistance. First and foremost, if resistance is defined as a genetic change leading to control failure in the *field*, then resistant strains should be both *field* derived and the costs of resistance should be studied in the *field*. Experiments on chronically selected resistant laboratory strains or on field collected strains tested in the laboratory, cannot really tell us much about likely fitness costs in the field. Second, the field collected strains that are compared should be both of known resistance genotype (homozygous susceptible *SS*, homozygous resistant *RR* or heterozygous *RS*) and should be compared in a similar genetic background (usually achieved by back-crossing resistance into a known susceptible background). Finally, if an experiment is conducted in the field, then ideally the resistant and susceptible strains should be competed directly against one another. If we apply these simple genetic criteria to the plethora of studies on fitness costs in the literature then very few studies pass all three of these tests. Therefore laboratory cage based competition studies showing, for example, a lack fitness cost associated with CYP6D1 mediated pyrethroid resistance in the house fly [6], need to be repeated under field conditions. In short the literature has therefore become a confusing array of studies conducted on a range of unrelated strains that may or may not have anything to do fitness costs in the field. Bearing all this in mind, it is now worth examining the few studies in which related strains or populations have been examined in the field.

One species where considerable efforts have been made to study resistance costs in well defined strains in the field is the Australian sheep blowfly, *Lucilia cuprina*. In this insect 70% mortality is observed in the overwintering (diapausing or developmentally arrested) larvae and diazinon resistant flies overwinter less successfully than their susceptible counterparts [7]. Critically, a ‘modifier’ locus of diazinon resistance has also been documented (see following discussion). When this modifier is restored to the resistant flies the overwintering success of resistant and susceptible flies is similar [7]. Similarly, dieldrin resistant (*Resistant to dieldrin* or *Rdl*) blowflies are also more strongly selected against during the Australian winter than at other times of the year [8]. These careful studies in the blowfly, which use genetically related susceptible and resistant strains with and without a fitness modifier, show us that the time of year in which field based fitness studies are performed is critical. Two further studies support the conclusion that overwintering can exacerbate the cost of resistance and that careful work studying resistance frequencies at all times of year are required. The first study examined the changes in resistance allele frequency of *Culex pipiens* mosquitoes overwintering in caves in the South of France. These mosquitoes carried two different resistance mechanisms either amplified esterases (termed as a single super locus, *Ester*) or altered acetylcholinesterase (encoded by *ace-1*). Whilst the changes in resistance frequencies observed can be altered by immigration of susceptible insects into the cave, changes in the frequency of *Ester* over the winter suggest that this super locus may be associated with a fitness cost as large as 42%. Similarly, a cost of 7% could be inferred for individuals that are homozygous resistant for *ace-1* or *ace-1^RR^* [9]. Finally, highly resistant clones of *Myzus persicae* aphids (clones R_2_ and R_3_) that over-express esterase-4 (E4), which can sequester and hydrolyse a range of insecticides. They show a reduced capacity to overwinter in the United Kingdom when compared to their susceptible (S) and moderately resistant (R_1_) counterparts [10].

## Mechanisms and modifiers

Even the most simplistic consideration of resistance mechanisms can give us a set of predictions about when and where mutation of a gene product might lead to a fitness cost. For target site resistance involving point mutations in so called ‘lethal’ genes encoding essential ion channel subunits, we would predict severe functional constraints on the nature and location of resistance associated mutations. The classic example of such constraints is shown by amino acid replacements in the GABA receptor subunit encoded by the *Rdl* gene. Here replacements of alanine301 both affect drug binding and also destabilise the drug preferred desensitised state of the receptor. Given this unique ‘dual’ resistance mechanism, nearly all insects showing cyclodiene resistance carry replacements of alanine301. In *Drosophila* at least, and in common with many other ion channel mutants, *Rdl-RR* flies show temperature sensitivity (paralysis at high temperatures) in comparison to their *SS* counterparts and like resistance this phenotype is also semi-dominant. However to our knowledge the effects of such temperature sensitive paralysis have not been investigated in the field for *Rdl* or indeed other target sites such as the *para* encoded sodium channel (*para^ts^* mutants were indeed originally isolated on this basis). Surprisingly however this narrow range of constraints does not apply to all ion channel subunits targeted by insecticides, despite the fact that these native ligand-gated ion channels are all composed of complex hetero-multimers of different ion channel subunits encoded by several different genes. Thus even native (rather than recombinant) GABA gated chloride ion channels containing *Rdl* encoded subunits are known to contain other subunits (despite the Rdl subunit alone conferring much of the insecticide relevant pharmacology). Thus a wide range of different mutations (including both point mutations [11, 12], exon-skipping [13^••^] or the production of truncated proteins [14, 15, 16]) can give rise to spinosad resistance in the α6 subunit of the nicotinic acetylcholine receptor. This is explained by the surprising finding that α6 knock-out strains of *Drosophila* are in fact not ‘lethal’ and also confer high levels of resistance to spinosad, leading the authors to speculate that ‘the viability of the mutant lacking the conserved Dα6 protein is striking, as is the lack of obvious fitness costs under laboratory conditions’ [17]. Again such surprising findings in the laboratory need to be tested under a range of field conditions.

**Figure 1:**
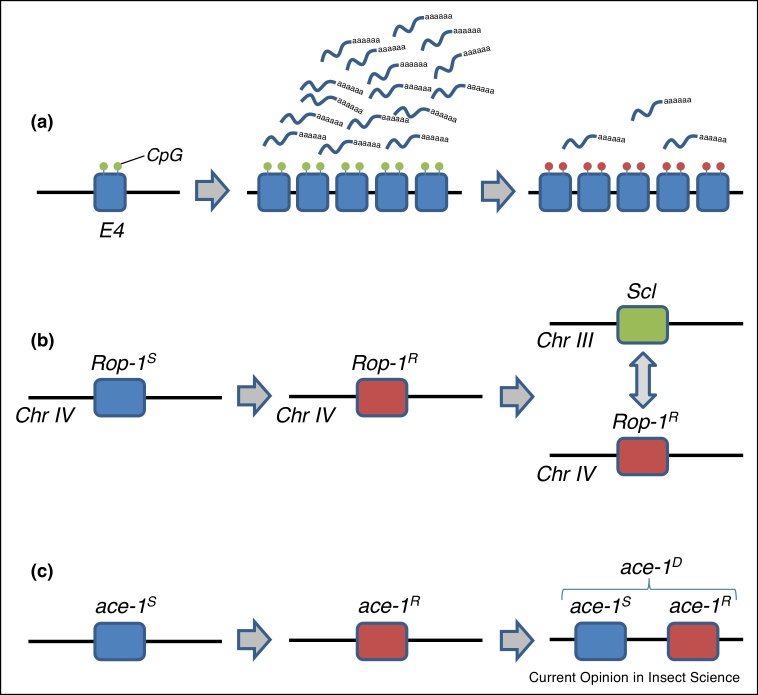
Mechanisms of overcoming fitness costs associated with insecticide resistance. **(a)** Differential methylation of amplified copies of esterase-4 (E4) in the aphid *Myzus persicae*. Gene amplification of E4 in highly resistant R_3_ clones (only five tandemly duplicated copies are shown here for simplicity) results in an increase in both the E4 transcript and E4 protein which can sequester and hydrolyse a range of organophosphorus and carbamate insecticides. However in the absence of insecticide, ‘revertant’ clones exhibit loss of both E4 expression and insecticide resistance. This loss of E4 expression is associated with loss of CpG sites within the amplified genes, resulting in gene silencing via demethylation. **(b)** Diazinon resistance evolution in the *Rop-1* locus encoding esterase-3 on chromosome IV of the Australian sheep blowfly *Lucilia cuprina* is followed by evolution of a fitness modifier *Scalloped wings* (*Scl*) on chromosome III. This fits the classical model for the evolution of a fitness modifier (see text for discussion). **(c)** Gene duplication leads to a compound heterozygote in which both a susceptible (*ace-1^S^*) and a resistant (*ace-1^R^*) copy of the *ace-1* gene are then found on the same chromosome. In this configuration any fitness costs associated with the *ace-1^R^* allele will be offset by the permanent presence of the susceptible allele along side it in permanent heterozygosis.

Similarly for metabolic resistance, if a resistance associated enzyme is ‘energetically’ costly to over-produce, then individuals over-expressing such an enzyme in the absence of pesticide would be at an energetic disadvantage. In fact, direct measurement of energetic resources (lipids, glycogen and glucose) in *Culex pipiens* mosquitoes over-expressing a resistance associated esterase suggests that resistant animals carry 30% less such energetic reserves than their susceptible counterparts [18], giving a clear mechanism for a potential fitness cost. In a second example, in the peach potato aphid, (*Myzus persicae*) over-expression of esterase-4 (E4) from tandemly repeated copies of the E4 gene might be seen as ‘costly’ in the absence of the pesticides that E4 metabolises or sequesters. However, rather unexpectedly, highly resistant (R_3_) clones of this aphid can switch off expression of these blocks of E4 genes using differential methylation [19, 20, 21]. In this manner, energetically costly over-production of E4 can be avoided in the absence of pesticide (Figure 1a). Importantly, once re-exposed to insecticide, selection within these ‘revertant’ clones can lead to increased levels of E4 expression again [21], although once again the relative fitness of these E4-revertant clones has not been tested in the field.

Classical theory suggests that if a *de novo* resistance gene carries a fitness cost, then other genes elsewhere in the genome might mutate to compensate for that cost and thus ameliorate the cost of resistance. As far as we are aware the only case where the molecular basis of such a modifier gene has been elucidated is in the case of the *Scalloped wings* (*Scl*) locus of *L. cuprina* (diagrammed in Figure 1b). *Scl* modifies the fitness of diazinon resistance conferred by esterase-3 (E3), which is encoded by the *Rop-1* locus [22]. The *Scl* locus is a homologue of the *Drosophila melanogaster Notch* gene and controls both fitness modification and fluctuating asymmetry [23], suggesting that it must also play a fundamental role in fly development. *Drosophila Notch* plays a role in determination of cell fate by mediating cell-cell interactions. Davies and co-workers speculated that the homologue *Scl* may therefore act by modifying the effects of the *Rop-1* esterase on cell adhesion during development [22]. Finally, before we leave the concept of fitness modifiers we need to recognise that different resistance genes themselves may also modify the overall fitness of the insect in which they are found. Such interactions have been hinted at by laboratory experiments with carbamate (*ace-1^R^*) and pyrethroid (*knock-down resistance* or *kdr*) resistant isogenic strains of *Culex quinquefasciatus* [24]. In these experiments the costs of harbouring both resistance genes were significantly less than those associated with *ace-1^R^* alone suggesting a significant interaction between the two genes. In the context of sexually reproducing insects, different alleles of different resistance loci will be shuffled during recombination. But in insects reproducing asexually, such as anholocyclic *M. persicae*, these fitness modifying combinations of resistance alleles may become locked in the same clone [10] thereby potentially favouring their spread or indeed decline.

## Pre-existing polymorphisms

All of the theory we have discussed till now assumes that all resistance associated mutations arise *de novo* after the introduction of the insecticide and, in the absence of a time-machine, this assumption has been widely taken for granted. However, once again, careful studies in Australian sheep blowflies have shown that this is not always the case. In a break-through study, Hartley and co-workers [25] took the ingenious step of using the Polymerase Chain Reaction (PCR) to look at the frequencies of mutations within esterase-3 (E3) causing either malathion or diazinon resistance. They looked at pinned blowflies collected before the introduction of organophosphorus insecticides (OPs) in 1950 and found no mutations conferring diazinon resistance but two of the twenty-one flies tested did carry the point mutation associated with malathion resistance. Whilst the sample numbers are small, this is ‘proof positive’ that malathion resistance pre-dated the introduction of malathion itself. This suggests that the malathion resistant E3 allele (containing the replacement Trp251Leu) may well have been a balanced polymorphism in the population prior to insecticide exposure, and this is consistent with the observed absence of a fitness cost in this allele which shows only a reduced carboxylesterase activity. In contrast, the diazinon resistant E3 allele (carrying the Gly137Asp replacement) must have occurred after the introduction of OPs and in turn this mutation not only reduces fitness but also completely abolishes carboxylesterase activity in E3. It is worth noting that these same two mutations in the house fly orthologue of E3 are also associated with resistance, and that replacement of the equivalent amino acid to Gly137 (Gly119 in other insects) decreases the sensitivity of acetylcholinesterase to insecticides in mosquitoes and even butyrylcholinesterase in humans (see Ref. [26] for a fuller discussion).

As these ‘pre-existing’ mutations would be expected not to carry a cost in the absence of pesticide (assuming that they were indeed ‘balanced’ polymorphisms prior to the advent of insecticides) it is important to try and determine how common they are in other forms of insecticide resistance. To date other studies that have used PCR to look for known resistance associated mutations in museum specimens are lacking, at least from the published literature. However other studies have examined if host–plant shifts have pre-adapted some insect strains to become resistant to insecticides via pre-existing ‘tolerance’ to host plant derived toxins. The best example of this is the evolution of a nicotine resistant form of the aphid *Myzus persicae*, termed *M. p. nicotianae*. In this subspecies, the ability to survive on tobacco plants is associated with a gene duplication of the nicotine metabolising P450 CYP6CY3 and an expansion of a dinucleotide repeat in the gene promoter of the P450 gene that also up-regulates P450 expression [27]. This same nicotine metabolsing P450 also by chance provides cross-resistance to insecticides the ‘neo’-nicotinoids (in fact stabilised nicotine mimics) [27] and thus this subspecies of aphid was already ‘resistant’ to these synthetic nicotine derivatives prior to their introduction via its pre-existing ‘tolerance’ to host plant nicotine.

## Adaptive walks and permanent heterozygosis

So how has our growing knowledge of the molecular biology of resistance informed the likely mechanisms behind any associated fitness costs? With the increased facility in genomic sequencing, we are seeing that many apparently simple mechanisms are in fact rather complex. The classic example of this is DDT mediated resistance in *Drosophila* conferred by over-expression of CYP6G1, which has been recently and comprehensively reviewed elsewhere [28^•^]. Initially the resistance allele was described as carrying 491 base pairs of DNA from the long terminal repeat of an *Accord* retrotransposon within the 5′ end of the *Cyp6g1* gene [29] which leads to constitutive over-transcription of the gene [30] and a strong selective sweep at this locus [31^••^]. However, a more comprehensive survey of extant resistance alleles has described the ancestral and susceptible *M* haplotype and the subsequent emergence of three highly resistant haplotypes (termed *AA*, *BA* and *BP*) that evolved via a series of steps involving gene duplication and multiple insertions of three different transposable elements. Each of these new alleles increases *Cyp6g1* transcription and thus increases resistance [32], in a so called ‘adaptive walk’. Whilst these new alleles improve the fitness of flies in the presence of insecticide it is interesting to ask if they also improve fitness in the absence of pesticide selection. The answer to this question remains largely unresolved. However, the original *Accord* insertion does show latitudinal variation in both the USA and Australia [33, 34], and different populations from across Australia show a strong genetic interaction between temperature and resistance [35^•^]. This suggests that temperature may play a role in determining the fitness of DDT resistant flies both in the presence and absence of pesticide, and that the fitness of the different resistance alleles at different temperatures may warrant further investigation.

A second fitness modifying concept that has only come to light recently is the concept of ‘permanent heterozygosis’. In one working of this example, a susceptible gene (*S*) becomes duplicated to form a second copy and this second copy (perhaps removed from its original functional constraints) becomes resistant (*R*). This duplication therefore leads to a compound heterozygote where both the *S* and *R* gene are physically linked on the same chromosome as *S–R* (Figure 1c). In this manner, the wild type function of the S gene is permanently preserved alongside the pesticide surviving capabilities of the new *R* allele. This *S–R* allele, carrying two different alleles, has been termed ‘heterogeneous’ [36^••^]. Alternatively, two *R* of the same alleles may become duplicated to form a ‘homogenous’ *R–R* duplication. This has recently and strikingly been illustrated in the *ace-1* gene of the malarial mosquito *Anopheles gambiae* where all 173 field collected mosquitoes analysed carried a duplication [36^••^]. Importantly, and fitting the above predictions, heterogeneous (*S–R*) duplications had intermediate phenotypes (lower resistance and fitness costs) whilst homogenous (*R–R*) duplications increased both pesticide resistance and fitness costs [36^••^]. It is however worth noting that both of these duplications form tandem 203 kilobase amplicons which also amplify 11 other genes [36^••^] and the fitness costs of over-expressing these extra genes currently remains unclear.

## Sexual antagonism

If resistance associated variation is to be maintained in the absence of pesticide selection then sexual selection may be one mechanism underlying such variation. The original finding that malathion resistant red flour beetles, *Tribolium castaneum*, had improved mating success over their susceptible counterparts [37] first led to the idea that carrying a resistance gene could actually benefit one sex or another. Other studies have found that heterozygous (*RS*) males of *Anopheles gambiae* had higher mating success than their homozygous resistant (*RR*) counterparts, both for *kdr* and *Rdl* mediated resistance. This suggests that being homozygous for target site resistance, in this case, carries a cost in reduced mating success. However we must return to the story of DDT resistance (*DDT-R*) conferred by the over-expression of CYP6G1 to gain a fuller understanding of the potential role of sexual conflict. Studies by McCart and co-workers surprisingly showed that, when inherited via the female, *DDT-R* increases adult fecundity, increases both egg and larval viability and speeds both larval and pupal development [38]. In contrast, further studies showed that *DDT-R* simultaneously reduces male fitness [39]. The *DDT-R* locus is therefore a rare documentation of a ‘sexually antagonistic’ locus which confers different fitness levels to the different sexes [40]. This may help explain why this resistance mechanism does not always spread to fixation in the absence of pesticide, despite its apparent benefit when inherited via the female [40]. In the future it will therefore be interesting to document differences in fitness costs between the sexes in order to see how widespread this sexually antagonistic maintenance of genetic variation may be.

## Gene editing and the future

**Figure 2:**
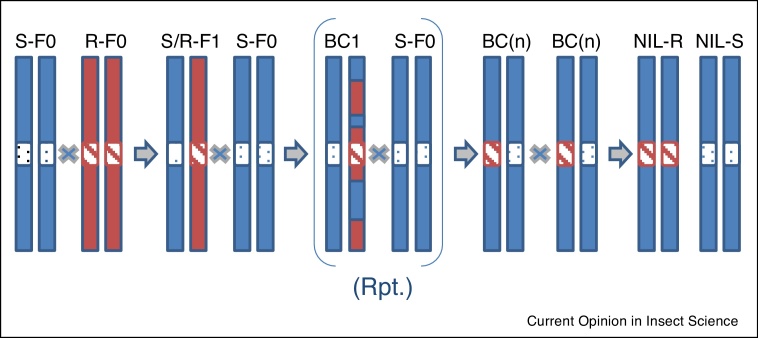
Backcrossing a resistance gene (R) into a susceptible (S) genetic background is a pre-requisite for proper fitness comparisons. However, several generations of backcrossing (BC) are necessary to completely replace the ‘resistant’ genome with the ‘susceptible’ one and generate near isogenic lines (NIL). Whilst easy to do in the fruit flies, the difficulty of backcrossing strains of pest insects has hampered our ability to properly compare fitness costs (see text for full discussion).

One reason why the susceptible and resistant strains compared in fitness studies do not share a common genetic background is that back-crossing is time consuming. It therefore takes a number of back-crosses to replace all of the resistant genome with its susceptible counterpart, thus leaving only the resistance associated mutation in the susceptible genome (Figure 2a). However given the recent explosion in CRISPR-CAS based gene editing it should now be possible to make resistance associated mutations directly within the genome of interest thus avoiding the need for extensive back-crossing. Such gene editing was first used to look at the effects of the P146S mutation in the *Drosophila* α6 subunit of the nicotinic acetylcholine receptor by placing it into a controlled genetic background [41]. Gene editing has since been used to look at a range of different resistance associated mutations in both *Drosophila* [42^••^] and, importantly, pest insects themselves [43••, 44•]. Similarly, simple gene knock-outs can be used to study potentially pleiotropic behavioural effects resulting from the loss of different ion channel subunits. For example, Somers and co-workers recently knocked-out the Dα1gene in a controlled genetic background and showed that loss of this subunit was associated with changes in courtship, sleep, longevity and insecticide resistance, revealing that a range of potential fitness costs might be associated with changes in this nicotinic acetylcholine receptor subunit [45^•^].

So where does this leave us for the future study of fitness costs? There is much still to be done, despite the advent of gene editing and the simplification of now introducing candidate resistance associated mutations into defined genetic backgrounds in pest insects, and our ability to make informed guesses about the nature of resistance costs (or their absence) from our increased knowledge of their molecular biology. Essentially once a resistance mutation is in the correct genetic background it still needs to be tested in the field and at a time of year when costs are most likely to be seen. Given that such experiments are long and difficult it is likely that future progress will continue to be slow. However what is clear is that if the costs of resistance are small or non-existent then resistance management strategies that rely on alternations will not work in the longer term. Therefore in the absence of a cost, resistance can only be overcome by the introduction of a new class of chemistry to which no pre-existing mechanisms confer cross-resistance. Thus, just as in the search for new antibiotics, the need for new classes of insecticide remains paramount.

## References and recommended reading

Papers of particular interest, published within the period of review, have been highlighted as:

• of special interest
•• of outstanding interest
